# Evaluation of High-Temperature Hydrogen Sensors Based on BaCe_0.6_Zr_0.3_Y_0.1_O_3-α_ and Sr(Ce_0.9_Zr_0.1_)_0.95_Yb_0.05_O_3-α_ Perovskites for Industrial Applications

**DOI:** 10.3390/s20247258

**Published:** 2020-12-18

**Authors:** Antonio Hinojo, Iván Soriano, Jordi Abellà, Sergi Colominas

**Affiliations:** Electrochemical Methods Laboratory—Analytical and Applied Chemistry Department, IQS School of Engineering, Universitat Ramon Llull, Via Augusta 390, 08017 Barcelona, Spain; antonio.hinojo@iqs.url.edu (A.H.); ivansorianoh@iqs.url.edu (I.S.); jordi.abella@iqs.edu (J.A.)

**Keywords:** proton-conducting ceramics, perovskite, amperometric, hydrogen sensor

## Abstract

In many industrial fields, there is a need to design and characterize on-line and on-board hydrogen monitoring tools able to operate under extreme conditions. One of these applications is in future nuclear fusion reactors, which will use hydrogen isotopes as a plasma fuel. In this context, the measurement of the concentration of these hydrogen isotopes will be of interest to ensure the correct operating conditions for such reactors. Hydrogen sensors based on solid-state electrolytes will be the first step in the development of new analytical tools able to quantify deuterium and tritium in aggressive environments. In the present work, amperometric hydrogen sensors were constructed and evaluated using two solid-state electrolytes, BaCe_0.6_Zr_0.3_Y_0.1_O_3-α_ and Sr(Ce_0.9_Zr_0.1_)_0.95_Yb_0.05_O_3-α_. Prototype sensors were built in order to study their sensitivity in on-line measurements. The experiments were performed in a reactor with a hydrogen-controlled environment. The sensors were evaluated at 500 and 600 °C in amperometric mode by applying 2 and 4 V voltages between electrodes. Both sensors showed increases in sensitivity when the temperature or voltage were increased.

## 1. Introduction

In many industrial fields there is a need to design and characterize on-line and on-board gas monitoring tools able to operate under high temperatures. A classic example is in the field of metallurgy, in which gas accumulation may result in residual tensions and cause breakage of the material core or negatively influence the processing [1,2,3]. One of these gases is hydrogen, which is usually controlled using off-line monitoring methods in high-temperature processes. 

An emerging field covering the development of new analytical tools able to quantify hydrogen and its isotopes is nuclear fusion technology. In such reactors, the use of deuterium and tritium is suggested as plasma fuels [4,5,6]. In this context, the development of specific probes to measure these isotopes will be of major interest and could provide experimental proof of the tritium self-sufficiency of these reactors.

The above-mentioned examples highlight a need in industry for the design and characterization of on-line and on-board hydrogen monitoring tools able to operate under extreme conditions. Solid-state proton conductors have attracted significant interest because of their chemical and physical durability, especially at elevated temperatures, and also their ability to extend the application to electrochemical hydrogen sensor systems for high-temperatures processes.

Perovskite crystal structure materials show proton conductivity typically in the range of 400–1000 °C [7]. Point defects in the lattice, such as oxygen vacancies, allow this phenomenon [8]. To increase these defects in the crystal structure, doping materials are commonly used. The addition of the dopants generates oxygen vacancies, which in the presence of water vapor or hydrogen, generate hydroxyl ions [9,10,11], as shown in Reaction (1).

(1) $$\frac{1}{2}H_{2}\left(g\right)+O_{O}^{x}\;⇄{\mathrm{OH}}_{O}^{•}+e^{\prime }$$

where $O_{O}^{x}$, ${\mathrm{OH}}_{O}^{•}$, and $e^{\prime }$ are an oxygen ion, a hydroxyl ion on the oxygen lattice site, and an electron, respectively. Then, following the Grotthuss mechanism [12], protons can be transported along the crystal structure. This conduction mechanism occurs due to conducting species, such as hydronium ions [13,14] or protons [8]. In this case, the conducting species are H^+^ ions, which are always located in the electronic cloud of an oxygen ion in the network. Thus, the Grotthuss mechanism can be decomposed into two steps. First, a proton in a hydroxyl ion is reoriented around the oxygen rapidly [15], then the proton jumps toward the nearest oxygen neighbour, creating a new hydroxyl [16]. 

Using this mechanism, electrochemical sensors based on proton-conducting, solid-state BaCe_0.6_Zr_0.3_Y_0.1_O_3-α_ and Sr(Ce_0.9_Zr_0.1_)_0.95_Yb_0.05_O_3-α_ electrolytes were designed and evaluated under different hydrogen concentrations. The effects of temperature and applied voltage on the sensors’ responses were evaluated and compared for both ceramics. 

## 2. Materials and Methods 

In previous works, different proton-conducting ceramics were evaluated [17,18,19,20,21]. From these studies, BaCe_0.6_Zr_0.3_Y_0.1_O_3-α_ (BaCeZrY) and Sr(Ce_0.9_Zr_0.1_)_0.95_Yb_0.05_O_3-α_ (SrCeZrYb) were selected as the most promising materials to be tested in future hydrogen sensors [18,19]. For this reason, the present work focuses on these two proton-conducting oxides (BaCeZrY and SrCeZrYb).

### 2.1. Synthesis and Sintering of Ceramics

The BaCeZrY ceramic was synthesized by solid-state reaction [22]. Briefly, stoichiometric quantities of powder reactants were mixed in a ball mill for 24 h: BaCO_3_ (Acros, Geel, Belgium, 99+%), CeO_2_ (Acros, 99.9%), 3%mol yttria-stabilized (YSZ, Sigma-Aldrich, St. Louis, MI, USA), Y_2_O_3_ (99.9%, Alfa Aesar, Ward Hill, MA, USA). Then, 20 g of powder mixture was suspended in 40 mL of ethanol (99.8%, Panreac, Barcelona, Spain) and ground in a planetary ball mill (RETSCH PM100) for 30 min at 350 rpm. Then, the mixture was dried and calcined for 24 h at 1400 °C. To ensure complete reaction, the mixing and calcination processes were repeated once.

The SrCeZrYb ceramic was synthesized using the citrate method [23,24,25]. Briefly, to synthetize 20 g of ceramic powder, stoichiometric quantities of the precursors were mixed and dissolved in 700 mL of deionized water: Sr(NO_3_)_2_ (Acros, 99+%), Ce(NO_3_)_3_ · 6H_2_O (Acros, 99.5%), ZrO(NO_3_)_2_ · xH_2_O (Sigma-Aldrich, technical grade), Yb(NO_3_)_3_·xH_2_O (Alfa Aesar, 99.9% (REO)). Then, a second solution of 700 mL of citric acid (Panreac, 99.5%) 0.6 M was also prepared in order to achieve a proper mixture 3:1 of citrates and metal ions. Then, both solutions were mixed and the pH was adjusted to 8 by adding ammonia (PanReac, 30% v/v.) An amorphous gel was obtained through heating and stirring. Finally, the product was dried and calcined at 1100 °C for 24 h.

Once both ceramic powders were synthetized, pellets of each ceramic were prepared. In both cases, 0.8 g of the ceramic powder was pressed in a 13 mm mould by applying 30 MPa pressure for 1 h. After this, BaCeZrY pellets were sintered at 1400 °C for 30 h and SrCeZrYb at 1300 °C for 12 h.

### 2.2. Sensor Construction

**Figure 1:**
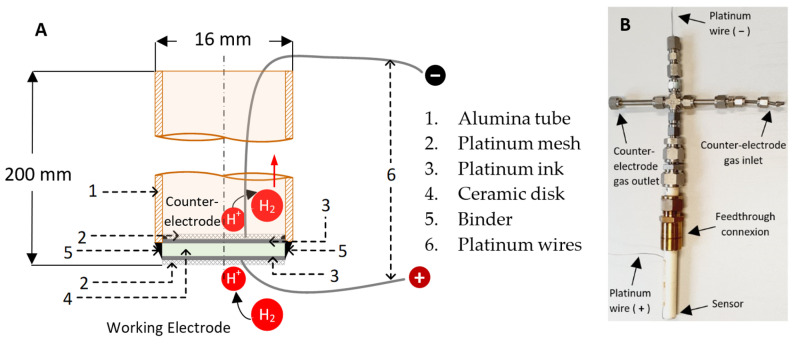
(**A**) Scheme showing the structure of the hydrogen sensor and (**B**) a picture of the sensor.

The sensors were constructed using alumina tubes (Al-23 Tube, both ends opened, OD 16 mm, ID 12 mm, 200 mm length, Alfa Aesar) and the sintered pellets of the solid-state electrolytes. Figure 1 shows a schematic representation of the hydrogen sensor. 

It can be seen in Figure 1A that the ceramic pellet was joined on one side of the alumina tube (Alfa Aesar,) using a binder composed of 45% regular glass, 35% aluminum oxide (Friatec Ceramics, 95%), and 20% sodium aluminum oxide (Alfa Aesar) dispersed within the smallest possible water volume in order to obtain a thick paste. The pellet was seated on it, dried, and heated up using a vertical tubular furnace at 1100 °C for 1 h.

Both sides of the pellet were platinized using a platinum ink (Alfa Aesar). In order to improve the electrical contact, platinum wires, which were used as positive and negative poles, were connected to a platinum mesh on both sides of the pellet (see Figure 1).

### 2.3. Electrochemical Measurements

**Figure 2:**
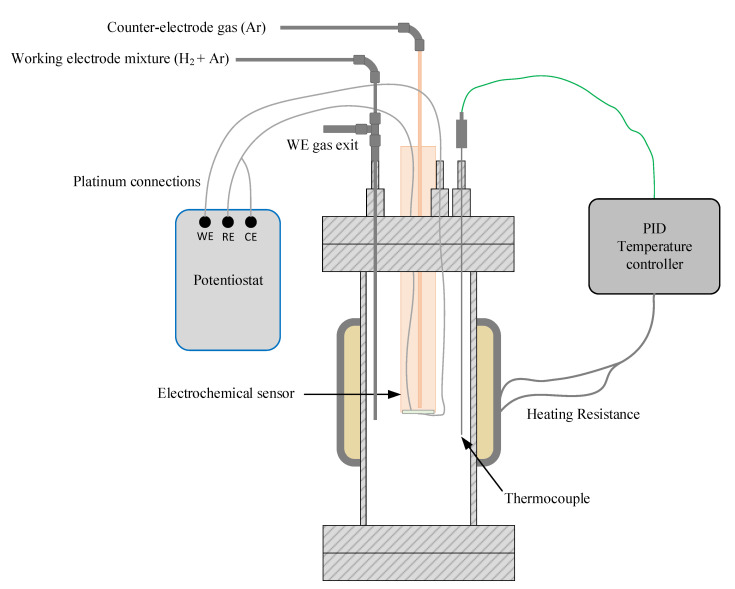
Representation of the reactor where the electrochemical measurements were performed, showing connections to external systems (WE: Working electrode, RE: Reference Electrode, CE: Counter-electrode).

Measurements were performed using a stainless steel reactor connected to a temperature controller unit. The external side of the platinized pellet was used as a working electrode (anode) and the inner side was used as a counter-electrode (cathode). Figure 2 shows a schematic representation of the reactor. 

As shown in Figure 2, the sensor was placed through the reactor cover using a feedthrough to avoid any gas leakage. The system was heated to the working temperature using a clamp-type electrical resistance (1500 W) and a PID-type (Proportional–Integral–Derivative) temperature controller (Fuji PXR4) with a K-type thermocouple. To reduce energy losses, the reactor was thermally isolated using a Kaowool™ insulator.

The hydrogen concentration was fixed or changed by mixing different flow rates of high-purity argon (99.9992%) and hydrogen calibration mixtures (0.5% H_2_ and 5% H_2_ in Ar), all of which were supplied by Carburos Metálicos. The flow rates were controlled using mass flow controllers (Bronkhorst EL-FLOW). In the working electrode compartment (anode), the gas mixture entered the system using a stainless steel tube through an inlet reactor port. In the counter-electrode (cathode), gas was injected on the inner chamber of the sensor using an alumina tube. The sensor’s platinum wires were connected to a PalmSens EmStat3+ Blue potentiostat–galvanostat to perform the electrochemical measurements.

Once the system was assembled at room temperature, the reactor was initially purged with high-purity argon to remove oxygen and after 1 h the temperature was raised to the working temperature (500 °C or 600 °C). Then, the desired hydrogen concentration was set and the current flowing through the electrodes was measured. In the working electrode (WE), a constant 500 mL/min flow rate was used, while in the counter-electrode (CE), 10 mL/min of high-purity argon was used. At each working temperature, measurements were performed by applying 2 V and 4 V between the WE and CE. This operational voltages for amperometric sensors were optimized in previous studies [20]. The response time of the sensor was calculated by fixing a stability criterion. This consisted of the time needed to obtain a response with a variability lower than 0.1% with respect to the average response measured in the last minute.

## 3. Results and Discussion

### 3.1. Characterization of the Proton-Conducting Electrolytes

**Figure 3:**
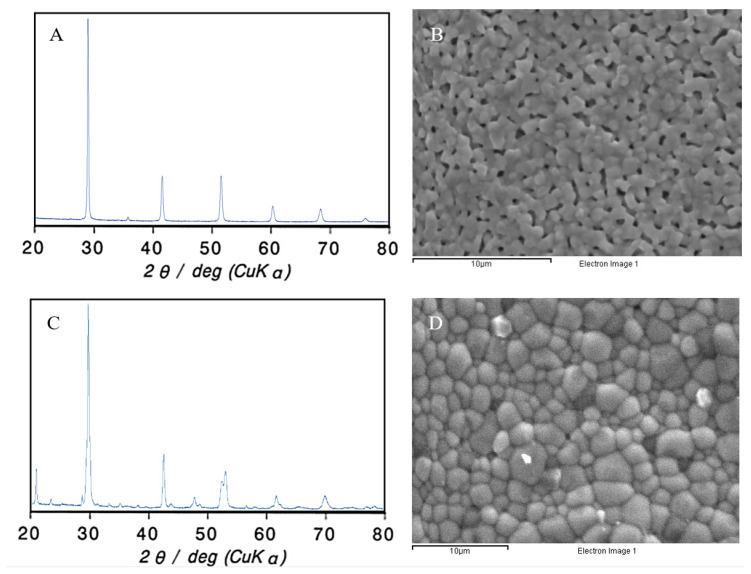
SEM and DRX characterization: (**A**) XRD pattern of BaCeZrY ceramic pellet; (**B**) sintered BaCeZrY ceramic pellet surface SEM micrograph; (**C**) XRD pattern of SrCeZrYb ceramic pellet; (**D**) sintered SrCeZrYb ceramic pellet surface SEM micrograph.

Ceramic pellets were characterized before the sensors were mounted. To this aim, XRD analysis was performed and compared with bibliographic results to verify the crystallographic structure. The instrument used was a Malvern Panalytical Empyrean using Cu Kα radiation. The surfaces of sintered pellets were also studied using an SEM electron microscope (JEOL JSM-5310). Figure 3 shows the XRD spectra and SEM micrographs of both electrolytes.

Figure 3A shows that a single perovskite phase was obtained when the BaCeZrY pellet was analyzed. The obtained spectra matched with data shown in the literature [26]. Figure 3B shows the morphology of the BaCeZrY pellet’s surface. No cracks were observed in the micrograph, despite the presence of some pores. For that reason, the pellet’s cross-section was analyzed using SEM to evaluate the presence of crossing channels (see supplementary material: Figure S1). The micrographs showed that there were few pores inside the pellet, but no preferential channels or cracks were observed. 

In Figure 3C it can be seen that the XRD spectra obtained for the SrCeZrYb ceramic matched the data in the literature [24]. There were no extra peaks, meaning that additional phases were not present in the material and a single perovskite-type structure was satisfactorily obtained. Figure 3D corresponds to a micrograph of the SrCeZrYb sintered pellet surface. As shown in Figure 3D, the ceramic surface had grains of different sizes, and that its surface was compact, dense, and without inconsistencies. No cracks, holes, or crossing channels were observed on the pellet surface.

### 3.2. Sensor Activation

**Figure 4:**
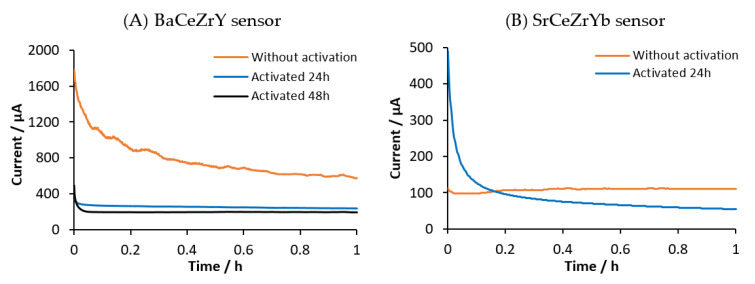
Comparison between the amperometric blank response without activation and activated (**A**) for 24 h and 48 h with the BaCeZrY sensor and (**B**) for 24 h with the SrCeZrYb sensor.

Perovskite-type conducting electrolytes are not only proton conductors but also ionic oxygen conductors [27]. This property can cause an offset in electrochemical measurements because oxygen competes with hydrogen for the ionic conduction. In order to remove oxygen trapped in the pellet structure after sintering, sensors were kept at 500 °C in a 5% hydrogen atmosphere (WE and CE) for 24 h to activate it. To verify the effectiveness of this process, the background current was measured before and after the activation process. Measurements were performed at 500 °C at an applied potential of 4 V (between WE and CE) and using a gas flow of high-purity argon at 500 mL/min in the WE and 10 mL/min in the CE. Figure 4 shows the background current measured after and before activation with both BaCeZrY and SrCeZrYb sensors. 

Figure 4A shows the background current in high-purity argon at different activation times (0, 24, and 48 h) using the BaCeZrY sensor. It can be observed (see Figure 4A) that the measured current of the sensor without activation at an applied voltage of 4 V after 1 h was 575 μA. After this initial measurement, the sensor was kept at 500 °C and at the open-circuit potential for 24 h in a 5% hydrogen (diluted in argon) environment at a constant rate of 50 mL/min in both compartments (WE and CE). Once this period had elapsed, measurements in a high-purity argon environment were registered again at 4 V. The blue line in Figure 4A shows that the measured current after 24 h activation was reduced to 239 μA. This means that the steady-state signal was reduced by 60% compared with the results obtained without activation. Finally, the sensor was kept again in 5% hydrogen for a new cycle of 24 h (for a total of 48 h of activation) and the blank measurement was repeated. In this case, the signal after 1 h was 198 μA. It can be seen that the reduction achieved after 48 h was considerably lower than the value obtained at 24 h (17% lower). 

Figure 4B shows the background current data obtained during the activation process using the SrCeZrYb sensor at 500 °C and at an applied voltage of 4 V between the WE and CE. Similar behaviour was observed as for the previous ceramic. When the measurement was performed without activation, the signal after 1 h was 111 μA. After 24 h in a 5% H_2_ atmosphere, the measurement in high-purity argon was 54 μA after 1 h. The signal after activation was reduced by 50% compared with the signal without activation. 

As shown in the above-mentioned experiments, an activation process must be performed for both ceramics. It was considered that 24 h was the proper period of time to activate the sensors. The oxygen source in the ceramics was attributed to air trapped inside the pellets during the shaping procedure [28]. The activation process using H_2_ fulfilled two functions. First, the displacement of trapped oxygen in the pellet, eliminating its contribution to the background current. At the same time, the material, which did not have intrinsic protons, incorporated them in the perovskite structure [29]. In this way, the electrolyte was ready to ionically conduct protons (as suggested by the Grotthuss mechanism [12,15,16,30,31,32,33,34]), ensuring that no oxygen contribution was obtained. 

### 3.3. Amperometric Measurements

#### 3.3.1. SrCeZrYb Electrolyte

**Figure 5:**
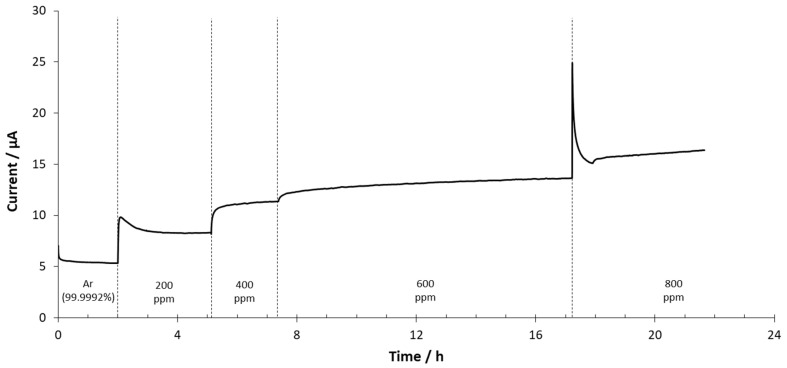
Chronoamperometry performed at 500 °C and 2 V with the SrCeZrYb sensor.

The first set of experiments was performed with the SrCeZrYb sensor at 500 °C and by applying 4 V between the electrodes. All measurements (see supplementary material: Figures S2–S5) were performed after the sensor’s activation. The WE total gas flow was 500 mL/min and 10 mL/min in the CE compartment. In both cases, high-purity argon was used as a covering gas to perform the blank measurement after activation. The H_2_ concentration in the anode (WE) ranged between high-purity argon (blank measurement) and 800 ppm of H_2_. The results are shown in Figure 5.

At the beginning of the experiment (see Figure 5), high-purity argon was injected into the reactor and the measured current became stable at around 5.3 µA, which was considered as the blank signal. When 200 ppm of H_2_ was injected, the response of the sensor increased to 8.2 µA. From 200 to 400 ppm, the signal increased to 11.3 µA, which represented a 3.1 µA increment. At 600 ppm, the current decreased to 13.6 µA (2.3 µA increment). Finally, at 800 ppm, the measured signal increased to 16.4 µA (2.8 µA increment). The transient state observed when the hydrogen concentration was changed could have been caused by local differences in the electrochemical activity along the overall surface of the pellet. The stabilization time of the mass flow controllers could also have contributed. The response time for the measurements was also evaluated. This parameter was calculated as the time needed to obtain a response variability lower than 0.1% with respect to average values from the last minute. The average value from 200 to 600 ppm was close to 15 min. 

**Figure 6:**
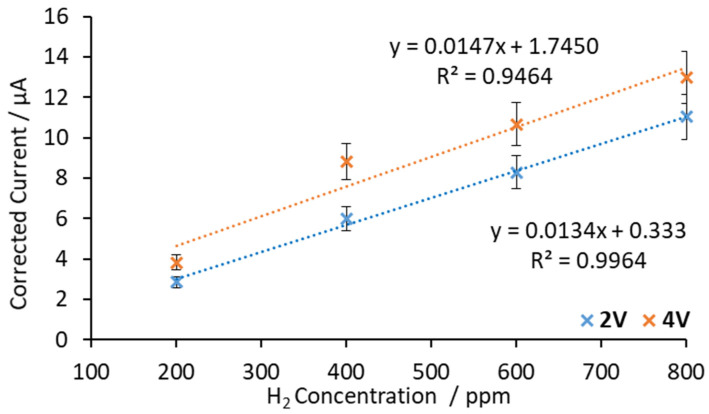
Calibration curves obtained with SrCeZrYb at 500 °C by applying 2 V and 4 V between the electrodes.

In order to analyze the sensor’s performance, the corrected current was represented as a function of the hydrogen concentration. The corrected current was calculated by subtracting the blank current from all measurements. The calibration curves obtained from the experiments at 500 °C by applying 2 and 4 V are both presented in Figure 6.

Comparing the obtained calibration curves at the same temperature (500 °C) when 2 V and 4 V were applied between the electrodes (see Figure 6), similar trends were observed. A small increase can be seen in the sensitivity between both voltages (10%). The slopes of the calibration curves were 0.0134 μA/ppm at 2 V and 0.0147 μA/ppm at 4 V. This phenomenon is explained by the increase in ion mobility caused by the change of the electric field. It can also be observed in Figure 6 that when 2 V was applied between the electrodes, the linear regression fit better than when 4 V was applied. 

**Figure 7:**
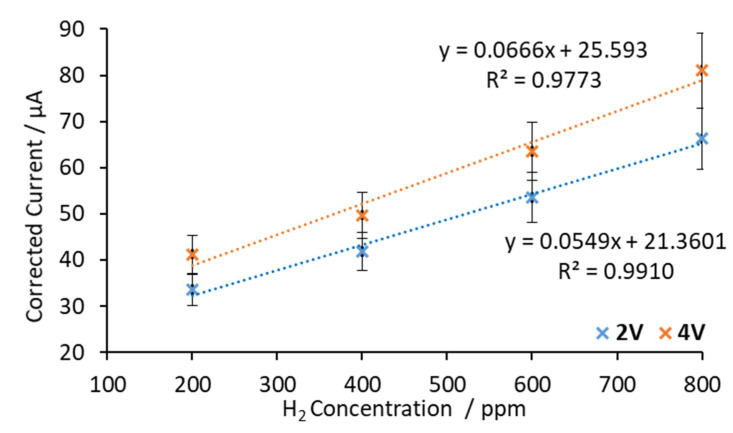
Calibration curves obtained with SrCeZrYb at 600 °C by applying 2 V and 4 V between electrodes.

In a second set of experiments the sensor response was measured at 600 °C. In this case, it was expected that the sensor response should increase. Perovskite-type materials are ion conductors due to oxygen vacancies in their structures. These point defects are dependent on temperature. At higher temperatures, more oxygen vacancies are formed, resulting in more ionic conductivity [35]. Figure 7 shows the corrected current vs. H_2_ concentration at 600 °C and voltages applied between electrodes of 2 V and 4 V.

As Figure 7 shows, linear trends were obtained and the slopes of the calibration curves were 0.0549 μA/ppm at 2 V and 0.0666 μA/ppm at 4 V. It can be seen that the sensitivity increased slightly when the applied voltage was changed from 2 V to 4 V (21%). However, similarly to the results at 500 °C, the results fit better in a linear regression model at 2 V than 4 V. 

In order to analyze the temperature effect on the sensitivity of the sensor, the obtained regression parameters are listed in Table 1 at both working temperatures (500 °C and 600 °C) at 2 V. Data obtained at 4 V were discarded for linear regression parameters (see Figure 6 and Figure 7). 

It can be seen in Table 1 that when the working temperature was increased from 500 °C to 600 °C, the sensor’s sensitivity increased. When the applied voltage was 2 V, an increment of 100 °C increased the sensitivity by a factor of 4. At 500 °C and 2 V, the sensor’s sensitivity was 0.0134 μA/ppm, while, at 600 °C it increased to 0.0549 μA/ppm. 

Note that the change of temperature from 500 °C to 600 °C implied an increase of proton carriers (${\mathrm{OH}}_{O}^{•}$) in the electrolyte lattice [36] and an increase of ion mobility [37]. Consequently, the ionic conductivity of the sensor increased. The increase of proton carriers implied increased intensity, and as a result increased sensitivity.

#### 3.3.2. BaCeZrY Electrolyte

**Figure 8:**
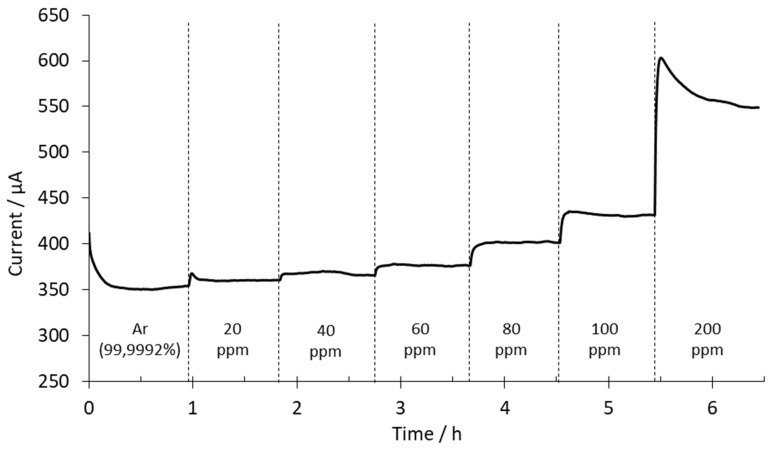
Chronoamperometry performed at 500 °C and 4 V with the BaCeZrY sensor.

In a new set of experiments, chronoamperometric measurements were performed at 500 °C and 4 V with the sensor based on the BaCeZrY ceramic. All measurements (see supplementary material: Figures S6–S9) were performed after the sensor’s activation. The WE total flow gas rate was set to 500 mL/min. Different mixtures of argon and hydrogen were used to change and fix the H_2_ concentration at the WE. Here, 10 mL/min of high-purity argon was injected in the CE compartment. The H_2_ concentrations used in the anode (WE) ranged between 0 and 200 ppm. From 0 to 100 ppm, the hydrogen content was increased in 20 ppm steps. The change from 100 to 200 ppm was done with a single step. Changes in hydrogen concentration were made after approximately 1 h. Figure 8 shows the registered current over time in these conditions.

At the beginning of the experiment, as shown in Figure 8, pure argon was injected in the reactor. The initial current was 412 μA, then after 1 h it stabilized at 354 μA. This value was considered as the blank signal. Then, the hydrogen content was increased inside the reactor to 20 ppm. The sensor response increased rapidly and stabilized at 360 μA. The signal increase obtained according to the blank signal was 6 μA. At 40 ppm, the response was 365 μA, which represented a 5 μA increase. At these low concentrations, the intensity was very similar to the background signal. At 60 ppm of hydrogen, the registered increments were higher after each hydrogen concentration change. At 60 ppm, the increment was 11 μA, at 80 ppm it was 25 μA, and at 100 ppm the intensity increased to 30 μA. After the stabilization of the signal at 100 ppm, the H_2_ content was increased to 200 ppm. This increment increased the signal from 431 μA to 548 μA (117 μA increment). 

As can be observed in Figure 8, the response obtained is a second-order system. For these types of systems, the characteristic parameters are the delay time (time required to reach 50% of the final value the very first time), rise time (time required to rise from 10 to 90% of the final value), and peak time (time required to reach the first peak of the overshoot). The parameters shown in Figure 8 are: delay times from 1 to 2 min, rise times from 0.7 to 4.7 min, and peak times from 2 to 5 min. In the other experiments performed with this ceramic, the values of these parameters were of the same order of magnitude.

The average response time for the sensor was 8 min (time needed to obtain a response variability lower than 0.1% with respect to average values from the last minute). In this case, from 20 to 100 ppm, the sensor needed 4 min to stabilize the signal. Note that the response time when the concentration was increased from 100 ppm to 200 ppm was 22 min. This change in the response time was attributed to the large change in the hydrogen concentration compared with the previous steps. 

**Figure 9:**
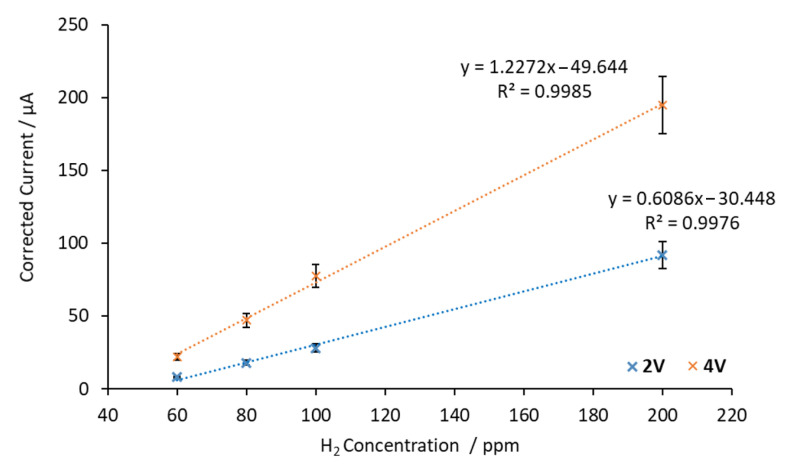
Calibration curves obtained with the BaCeZrY sensor by applying 2 V and 4 V between the electrodes at 500 °C.

In order to analyze the trend of the sensor response as a function of the hydrogen concentration, the corrected current vs. the H_2_ concentration was represented. Figure 9 shows the corrected currents as a function of the H_2_ concentration at 500 °C by applying 2 V and 4 V between electrodes. 

As shown in Figure 9, data obtained at H_2_ concentrations lower than 60 ppm were discarded. These points were too similar to the background current, which induced a loss of linearity in the sensor response. As a result, 60 ppm was considered as the minimum hydrogen concentration that the sensor was able to quantify.

Furthermore, significant differences can be noted when 4 V and 2 V currents were applied between the electrodes. When the applied voltage was 4 V, larger current values were obtained. As with the SrCeZrYb sensor, this phenomenon is explained by the increase in ion mobility caused by the change of the electric field.

The sensitivity obtained at 2 V was 0.6086 μA/ppm, while at 4 V it was 1.2272 μA/ppm. This means a sensitivity increment of 102% occurred when the applied voltage was changed from 2 V to 4 V. It is worth mentioning that at both voltages, the same linear concentration range was obtained (60–200 ppm). Finally, the average time needed to stabilize the signal after each H_2_ concentration change below 100 ppm when 2 V was applied was 6 min. This result was of the same order of magnitude as that obtained when 4 V was applied (4 min below 100 ppm). 

**Figure 10:**
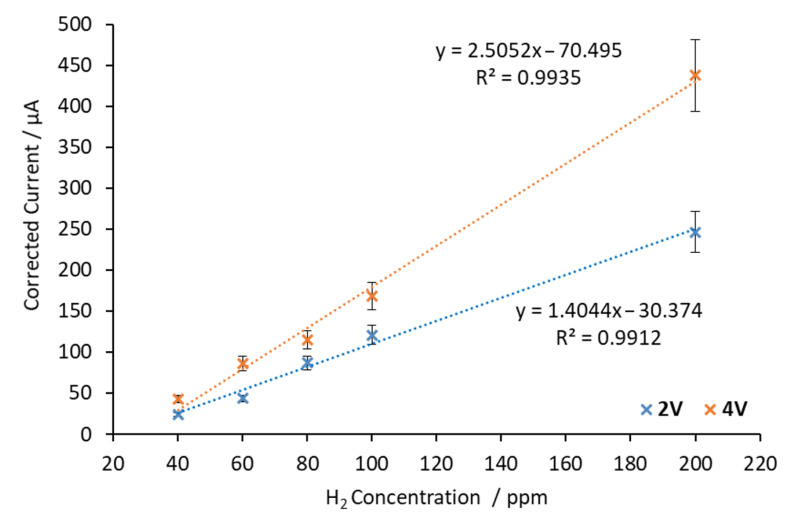
Calibration curves obtained with BaCeZrY by applying 2 V and 4 V between electrodes at 600 °C.

The same calibration procedure was repeated at 600 °C. The corresponding calibration curves are shown in Figure 10. 

It can be seen in Figure 10 that the sensor response obtained at 600 °C was very similar to that obtained at 500 °C (see Figure 6). After adjusting the data using linear regression, the obtained sensitivity values were 1.4044 μA/ppm at 2 V and 2.5052 μA/ppm at 4 V. This means a sensitivity increment of 78% occurred when the applied voltage was changed from 2 V to 4 V. The linear concentration range was 40–200 ppm at both applied voltages. The average response time below 100 ppm was around 5 min at both applied voltages.

In order to analyze the temperature effect on the sensitivity of the sensor, the regression parameters as well as the response time are shown in Table 2 at both working temperatures (500 °C and 600 °C) and applied voltages (2 V and 4 V).

Table 2 shows that when the applied voltage was 2 V at 500 °C, the sensor sensitivity was 0.6086 μA/ppm, while at 600 °C it increased to 1.4044 μA/ppm. It can be observed that an increase in the working temperature implies an increment in the sensor sensitivity. In this situation (applied voltage of 2 V), an increment of 100 °C increased the sensitivity by a factor close to 2. The same effect was observed when the applied voltage was 4 V. At 500 °C, the sensor sensitivity was 1.2272 μA/ppm, whereas at 600 °C it increased to 2.5052 μA/ppm. In this situation (applied voltage of 4 V), an increment of 100 °C increased the sensitivity by a factor of 2. As was the case when using SrCeZrYb, this fact was attributed to the increase in the ionic conductivity due to the increment in the working temperature.

The variability of both the SrCeZrYb and BaCeZrY sensors was analyzed. In both cases, this parameter was around 10%, indicating the good reliability of these devices, considering that they are in the development stage.

### 3.4. Comparison with Other Sensors Reported in the Literature

The obtained results were compared with other hydrogen sensors reported in the literature with similar conditions [38,39]. Table 3 shows the analytical parameters of the hydrogen sensors presented in this work and others found in the literature. 

It can be seen in Table 3 that the sensors presented in this work showed a linear relationship between the current and hydrogen concentration in the low hydrogen partial pressure range (approximately 40–200 ppm). Note that this concentration range is smaller than the results shown by other authors in similar working conditions (from thousands of ppm to tens of thousands ppm, see Table 3). It is worth mentioning that the linear concentration range of the sensors presented in this work is close to the expected tritium concentrations of future breeding systems (700 ppm [40]). This fact gives the sensors a great chance of being used in breeding blankets in future nuclear fusion reactors.

It may also be worth discussing the fact that the developed sensors had higher sensitivities than other sensors in the literature (see Table 3). For example, La_0.95_Sr_0.05_YO_3-α_ at 800 °C and 0.75 V had a sensitivity of 0.2085 μA/ppm [39]; in contrast, the BaCeZrY sensor in the worst case scenario (500 °C and 2 V) showed a value of 0.6086 μA/ppm, which is three times higher than the La_0.95_Sr_0.05_YO_3-α_ sensor. In addition, it was also demonstrated that the sensitivities of the sensors presented in this work increased when increasing the working temperature or applied voltage. This fact gives the sensors high versatility. In other words, it is possible to tune the analytical parameters of the sensors by changing the temperature or applied voltage.

## 4. Conclusions 

SrCeZrYb and BaCeZrY ceramics were satisfactorily synthetized and sintered. The activation process used for the sensors (24 h at 500 °C in 5% H_2_ in argon) decreased the blank signal by about 50–60%. This fact was attributed to the presence of trapped oxygen in the pellets during the shaping process, which caused an offset in amperometric measurements due to competition with hydrogen for the ionic conduction. 

The SrCeZrYb sensor was evaluated at 500 and 600 °C by applying 2 and 4 V between the electrodes. At the same voltages, sensitivity increases were observed when the temperature was increased. At 2 V, the sensitivities were 0.0134 μA/ppm at 500 °C and 0.0549 μA/ppm at 600 °C. Nevertheless, the operability range was lower than with the BaCeZrY sensor, because at 4 V the data did not fit properly with the linear regression model. 

The BaCeZrY sensor showed a sensitivity increase when the temperature or voltage were raised, with values between 0.61 µA/ppm (2 V and 500 °C) and 2.51 µA/ppm (4 V and 600 °C) being obtained. The relationship between sensitivity, temperature, and voltage makes this sensor a very versatile analytical tool, because its operational parameters can be tuned by modifying the working conditions.

Both sensors were demonstrated to be valid for the final application. On the one hand, the SrCeZrYb sensor showed linearity in the required range (200–800 ppm). On the other hand, the BaCeZrY sensor, which showed better sensitivity results, had a lower operation range. Nevertheless, this fact could be solved by diluting the samples to obtain a final concentration within the sensor’s range. In future studies, ceramic powders will be shaped using other geometries, which will increase the surface area in order to improve the electrochemical response.

## Figures and Tables

**Table 1:** Sensitivity results obtained from chronoamperometry tests with the SrCeZrYb sensor at 2 V and at 500 °C and 600 °C.

Operational Conditions	Sensitivity/μA·ppm^−1^	Range/ppm	Response Time/min
500 °C—2 V	0.0134	200–800	15
600 °C—2 V	0.0549	200–800	8

**Table 2:** Sensitivity results obtained from chronoamperometry tests with the BaCeZrY sensor at 500 °C and 600 °C and at 2 V and 4 V.

Operational Conditions	Sensitivity /μA·ppm^−1^	Range/ppm	Response Time/min
500 °C—2 V	0.6086	60–200	6
600 °C—2 V	1.4044	40–200	5
500 °C—4 V	1.2272	60–200	4
600 °C—4 V	2.5052	40–200	4

**Table 3:** Analytical parameters of the hydrogen sensors developed in this work and those found in the literature.

Reference	Electrolyte	Operational Conditions	Sensitivity/μA·ppm^−1^	Range/ppm
[38]	La_0.9_Sr_0.1_YO_3-α_	550 °C—2 V	0.0077	1000–33,000
[39]	La_0.95_Sr_0.05_YO_3-α_	800 °C—0.75 V	0.2085	5000–40,000
This work	Sr(Ce_0.9_Zr_0.1_)_0.95_Yb_0.05_O_3-__α_	500 °C—2 V	0.0134	200–800
		600 °C—2 V	0.0549	200–800
This work	BaCe_0.6_Zr_0.3_Y_0.1_O_3-α_	500 °C—2 V	0.6086	60–200
		600 °C—4 V	2.5052	40–200

## Supplementary Materials

The following are available online at https://www.mdpi.com/1424-8220/20/24/7258/s1.
