# Supplementary material for: Evaluation of High-Temperature Hydrogen Sensors Based on BaCe0.6Zr0.3Y0.1O3-α and Sr(Ce0.9Zr0.1)0.95Yb0.05O3-α Perovskites for Industrial Applications
Source: Sensors (Basel). 2020 Dec 18;20(24):7258. doi: 10.3390/s20247258 (PMC7766507; doi:10.3390/s20247258)
Supplement: Supplementary file 1 [file sensors-20-07258-s001.pdf]

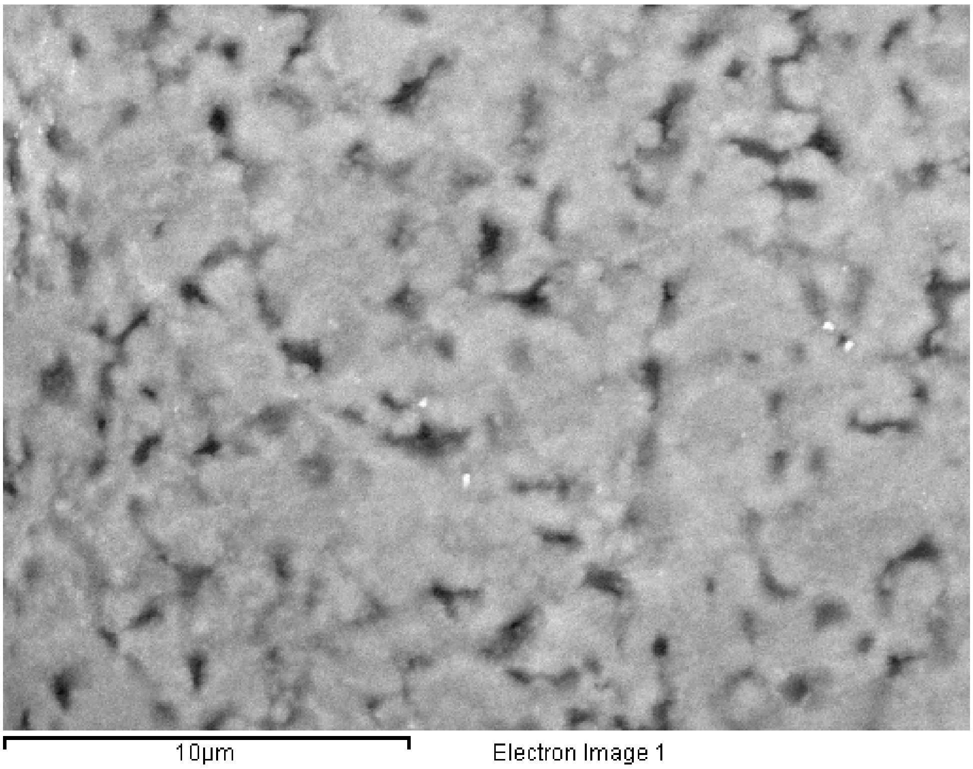

Figure S1. Micrograph of the BaCeZrY sintered disk section.

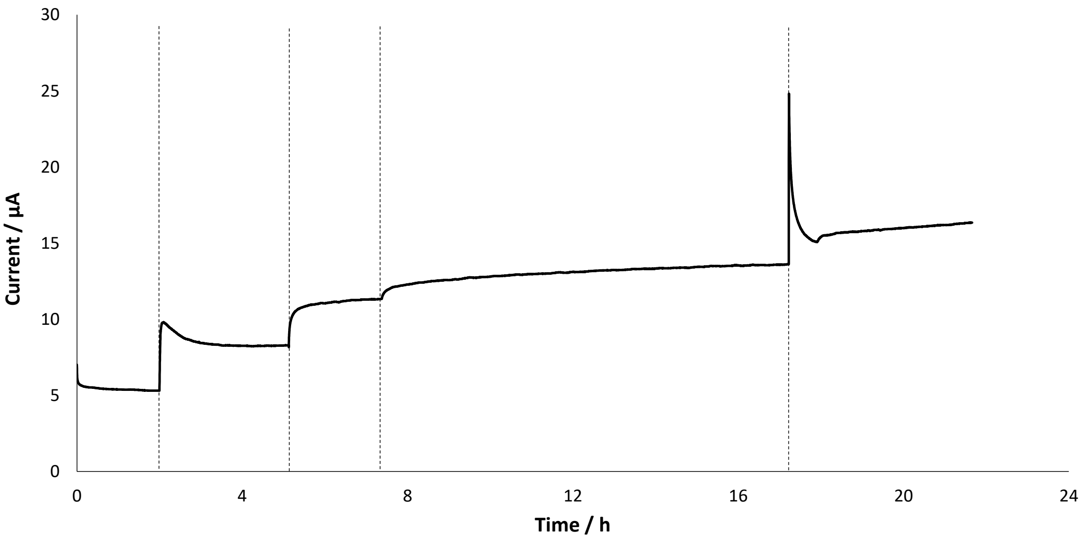

Figure S1. Chrono-amperometry obtained with SrCeZrYb sensor at 500°C and applying 2V between electrodes.

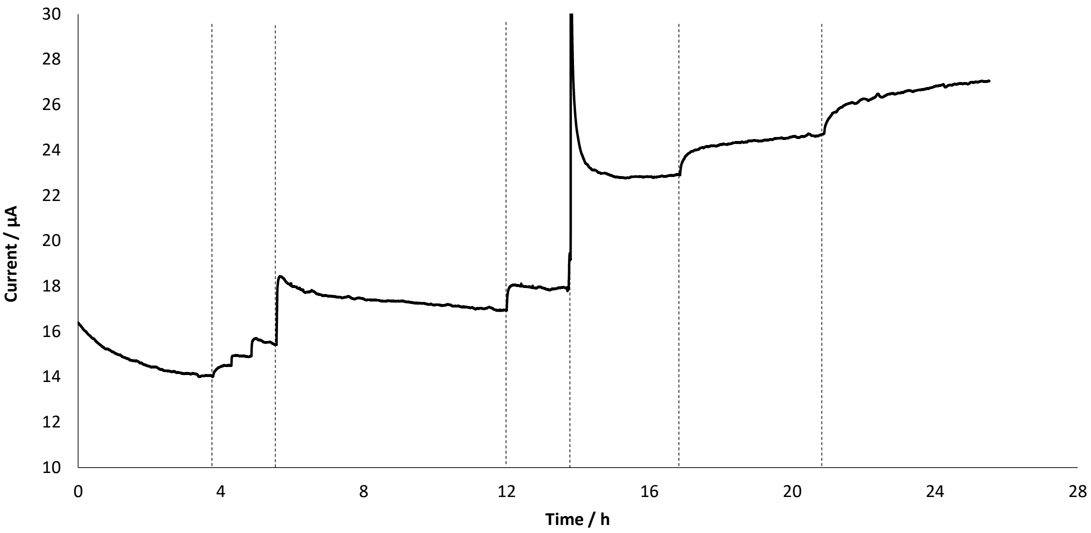

Figure S2. Chrono-amperometry obtained with SrCeZrYb sensor at 500°C and applying 4V between electrodes.

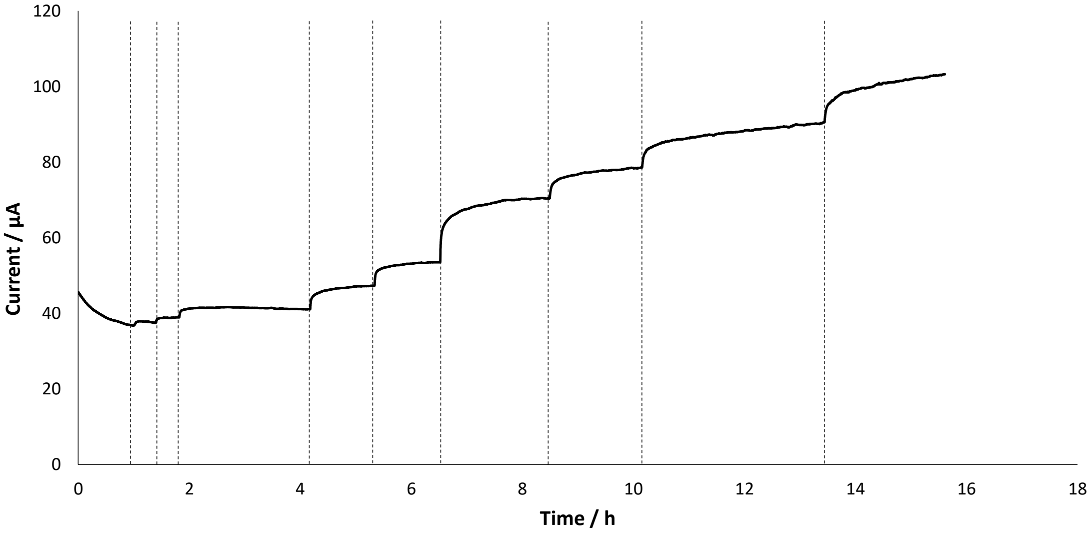

Figure S3. Chrono-amperometry obtained with SrCeZrYb sensor at 600°C and applying 2V between electrodes.

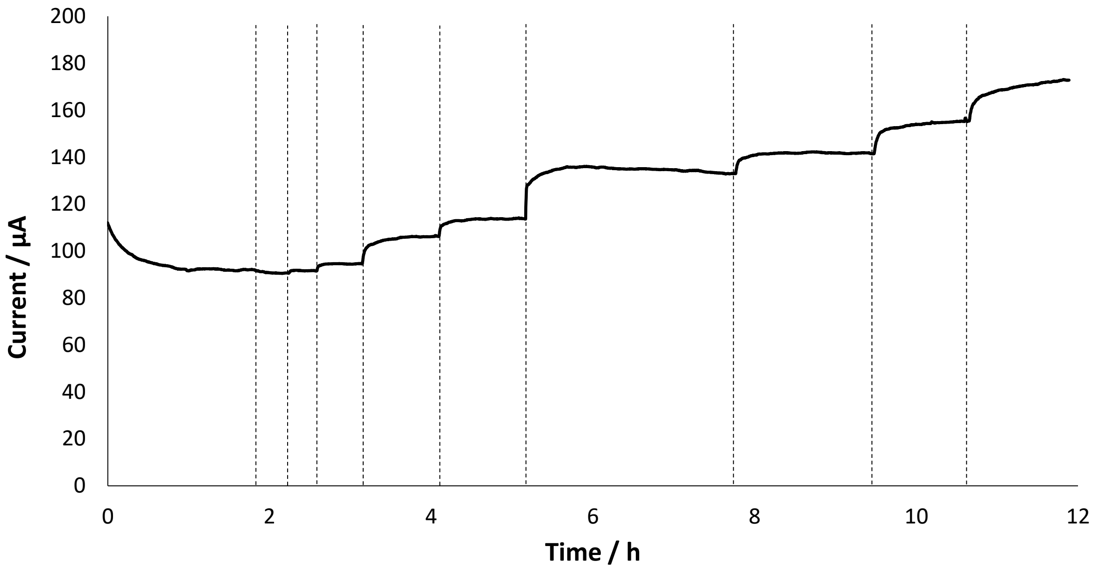

Figure S4. Chrono-amperometry obtained with SrCeZrYb sensor at 600°C and applying 4V between electrodes.

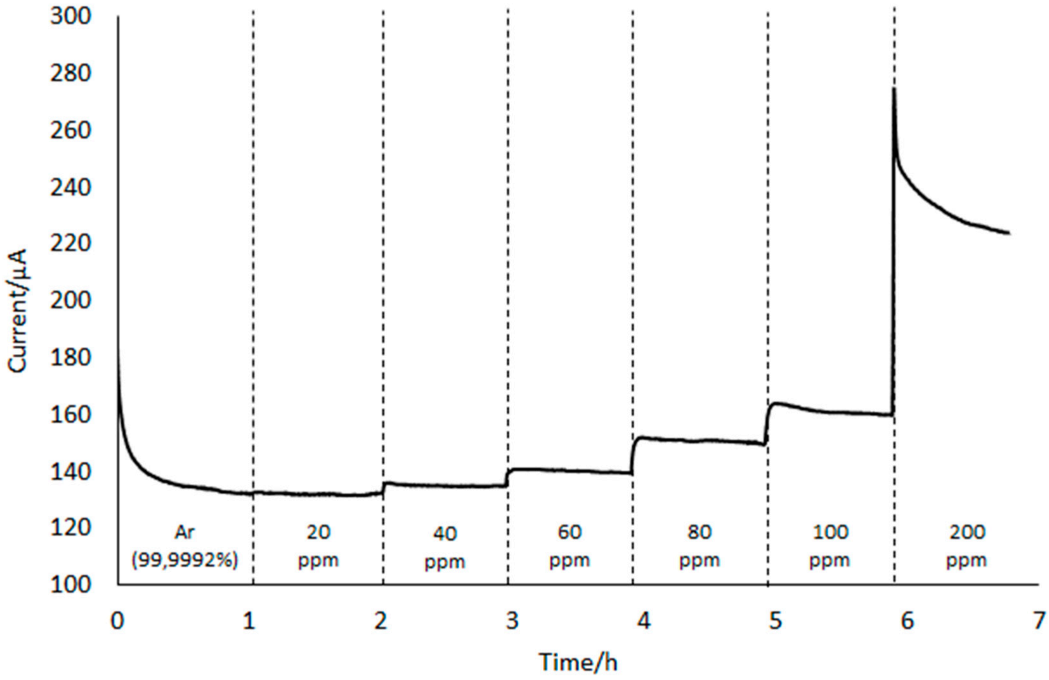

Figure S5. Chrono-amperometry obtained with BaCeZrY sensor at 500°C and applying 2V between electrodes.

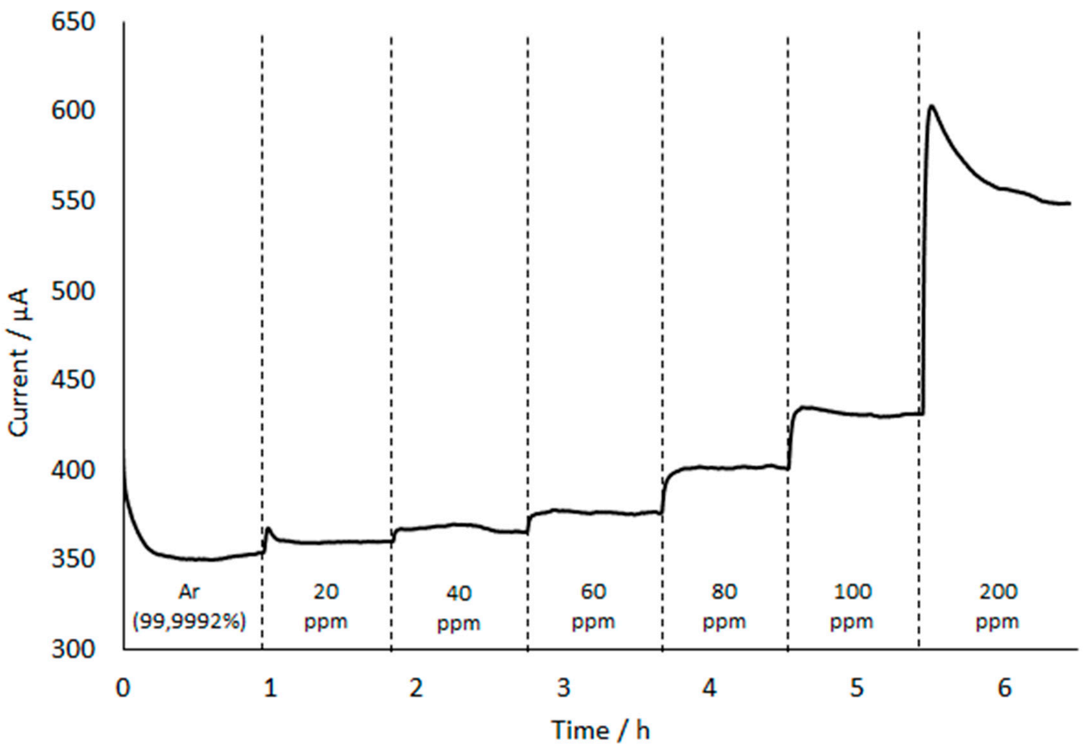

Figure S6. Chrono-amperometry obtained with BaCeZrY sensor at 500°C and applying 4V between electrodes.

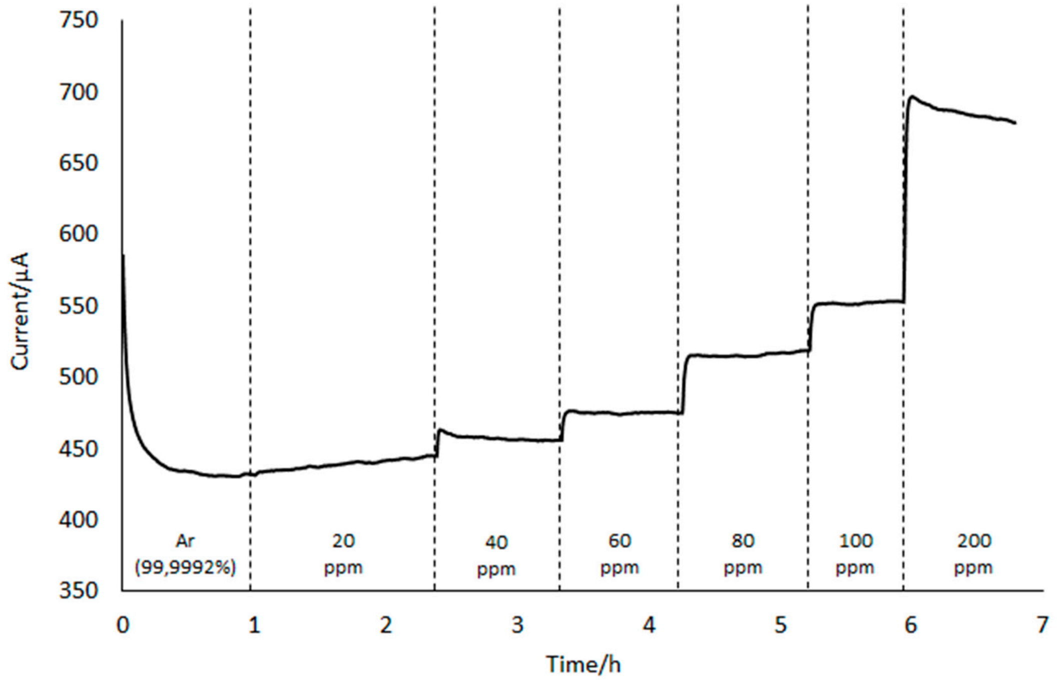

Figure S7. Chrono-amperometry obtained with BaCeZrY sensor at 600°C and applying 2V between electrodes.

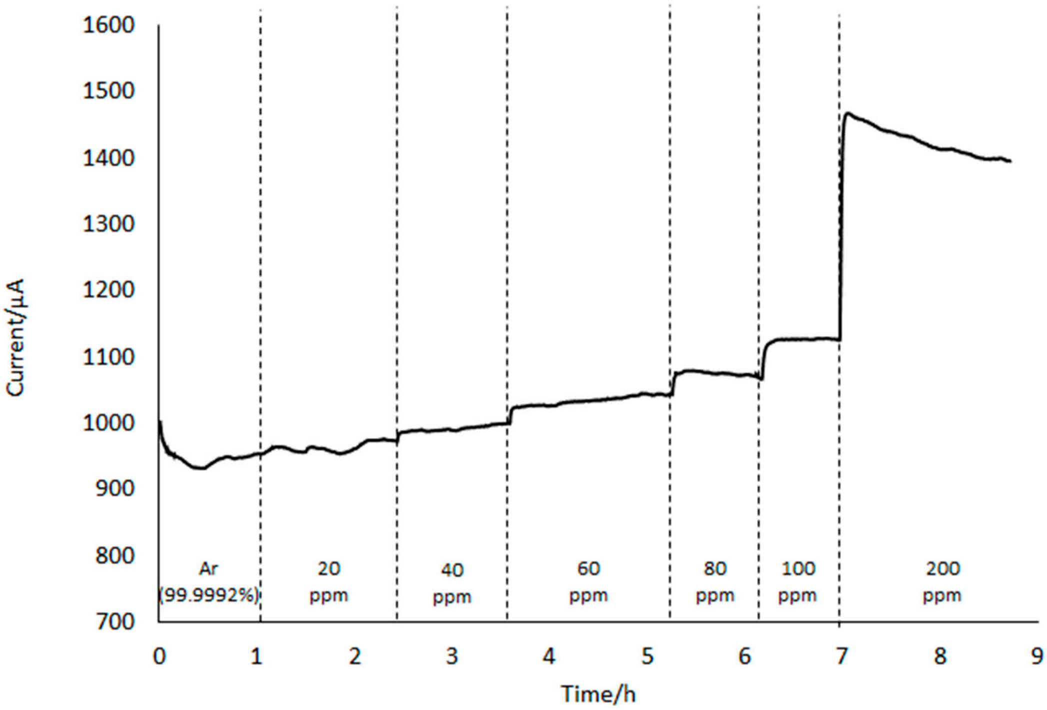

Figure S8. Chrono-amperometry obtained with BaCeZrY sensor at 600°C and applying 4V between electrodes.
